# Electrospun Polycaprolactone/Carbon Nanotube Membranes for Transdermal Drug Delivery Systems

**DOI:** 10.3390/polym18010015

**Published:** 2025-12-21

**Authors:** Elizabeth Ortiz-Maldonado, Eduardo San Martin-Martínez, Ningel Omar Gama-Castañeda, Marquidia Pacheco, Ulises Figueroa-López, Andrea Guevara-Morales, Esmeralda Juárez, Andy Ruiz, Horacio Vieyra

**Affiliations:** 1 Tecnologico de Monterrey, Escuela de Ingeniería y Ciencias, Eduardo Monroy Cárdenas 2000, San Antonio Buenavista, Toluca de Lerdo 50110, Mexico; a01368747@tec.mx; 2 Centro de Investigación en Ciencia Aplicada y Tecnología Avanzada, Instituto Politécnico Nacional, Legaria 694, Colonia Irrigación, Ciudad de México 11500, Mexico; esanmartin@ipn.mx (E.S.M.-M.); omar.gama06@hotmail.com (N.O.G.-C.); 3 Instituto Nacional de Investigaciones Nucleares, Carretera México Toluca, Ocoyoacac 52750, Mexico; marquidia.pacheco@inin.gob.mx; 4 Tecnologico de Monterrey, Escuela de Ingeniería y Ciencias, Carretera Lago de Guadalupe Km 3.5, Colonia Margarita Maza de Juárez, Atizapán de Zaragoza 52926, Mexico; ufiguero@tec.mx (U.F.-L.); a.guevaram@tec.mx (A.G.-M.); 5 Laboratorio de Alta Contención Biológica, Instituto Nacional de Enfermedades Respiratorias Ismael Cosío Villegas, Calz. de Tlalpan 4502, Sección XVI, Tlalpan, Ciudad de México 14080, Mexico; ejuarez@iner.gob.mx (E.J.); andy.ruiz@iner.gob.mx (A.R.)

**Keywords:** polycaprolactone, carbon nanotubes, electrospinning, membranes, biomedical membranes

## Abstract

The development of membranes and patches for controlled drug release to enhance therapeutic efficacy is a promising approach to addressing the challenge posed by poor adherence to pharmacological therapies for chronic diseases. In this study, we designed an electrospun polycaprolactone (PCL) nanofibrous membrane reinforced with different concentrations (0.04%, 0.05%, 0.075%, and 0.2%) of functionalized multi-walled carbon nanotubes (f-MWCNTs) intended for biomedical applications, such as transdermal devices. We characterized the resulting composites using scanning electron microscopy (SEM), Fourier-transform infrared spectroscopy (FTIR), atomic force microscopy (AFM), and dynamic mechanical analysis (DMA) to evaluate their morphology, chemical composition, and mechanical properties. We also measured their cytotoxicity upon contact with peripheral blood mononuclear cells. The nanofibers had diameters below 100 nm and inclusions of microspheres, which were attributed to the electrospinning expansion phenomenon. Spectroscopic and mechanical analyses confirmed molecular interactions between the PCL matrix and the f-MWCNTs. Finally, biological tests demonstrated that both the dispersion of f-MWCNTs and the nanofiber sizing render the membranes biocompatible, supporting their potential use as drug-delivery systems.

## 1. Introduction

Patient nonadherence to drug treatments for chronic diseases undermines treatment success. Patches for transdermal drug delivery are a possible solution to this problem. Polymer nanofibers and carbon nanotubes are promising drug carriers [1,2,3,4] that can be used to produce dermal patches that promote the controlled release of drugs, optimizing the effectiveness of medical treatments and overcoming the inconveniences of pills and injections. In this study, we combined the therapeutic advantages of both polymer nanofibers and carbon nanotubes.

Polycaprolactone (PCL) is a biodegradable polymer, compatible with biological systems, making it an optimal choice for various biomedical and environmental applications. It is obtained by ring-opening the lactone, linking the e-caprolactone monomers to form the PCL polymeric chains in a process called ring-opening polymerization (ROP) [5,6]. PCL is a semi-crystalline polymer with a low glass transition temperature of around −60 °C, making it strong and flexible [7]. PCL can be fashioned into sub-micron particles, thin films, melt-extruded matrices, and homopolymers/copolymers, with performance that depends on formulation design and drug properties for bone and cartilage applications [8,9,10]. Depending on the design, PCL may improve tissue uptake of lipophilic compounds, increase the permeation rates of certain drugs, involve intercellular lipids and hair follicles as primary routes of skin penetration, and limit drug accumulation in the stratum corneum [11,12,13].

In general, polymers have limitations in terms of rigidity, resistance, and thermal stability that may be overcome by reinforcing them with materials such as nanoparticles, fibers, and even other polymers [14]. PCL’s ability to be mixed with other materials, improving mechanical and thermal properties, such as degree of crystallinity, solubility, degradation, Young’s modulus, and tensile strength, makes it an ideal material for the manufacture of composites [15,16,17]. In this study, we are incorporating carbon nanotubes into the membrane design.

Carbon nanotubes are concentric carbon nanostructures that resemble graphene; they may occur as multi-walled (MWCNTs) or single-walled (SWCNTs), depending on their number of layers [18,19]. Carbon nanotubes are stable nanostructures that can be used for composites. This requires a surface modification known as functionalization, which involves linking functional groups and adsorbing functional molecules to the sidewalls and the internal cavity of carbon nanotubes [20], thereby dispersing them and preventing agglomeration, guaranteeing an optimal matrix-reinforcement interface when producing composite materials [21].

The main challenge of using carbon nanotubes in biomedicine is their interaction with cells, which raises concerns about their toxicity. The metal catalysts used in their synthesis, along with their nanoscale structure, can elicit a negative cellular response, such as oxidative stress [22,23]. Furthermore, the lack of understanding of how nanotubes enter cells and exert their potential toxic effects complicates their study and safe application [24]. Functionalization of carbon nanotubes modifies their chemical properties, reducing toxicity [25], and making them suitable for medical applications such as bioimaging, cancer treatment, drug delivery, and biosensors [26,27].

In this study, we used electrospinning to fabricate polycaprolactone (PCL) nanofibrous membranes. Electrospinning is a process that produces fibers and nanofibers from polymeric solutions by applying a high-voltage electric field, generating materials with unique nanoscale characteristics [28] that are functional for biomedical applications and the development of medical devices [29,30]. Currently, electrospun materials are used in the biomedical field as scaffolds for tissue engineering, drug delivery systems, and pharmacological membranes, designed to deliver drugs in a controlled manner directly into the wound or through the skin [31,32].

The PCL membranes were reinforced with different concentrations of functionalized multi-walled carbon nanotubes (hereafter referred to as MWCNTs) ranging from 0.04% to 0.2% for potential biomedical applications. The MWCNTs were functionalized via acid treatment to introduce carboxyl groups on their surfaces, thereby enhancing their dispersion and interfacial interactions with the polymer matrix [33]. The effect of MWCNT content on the mechanical, physical, and chemical properties of the composite membranes was systematically investigated [34,35]. Also, since the membrane fabrication process involves the use of organic solvents and chemical reagents, the safety of the resulting materials was assessed using peripheral blood mononuclear cells (PBMCs) from healthy donors to evaluate membrane biocompatibility [36,37]. Our results demonstrated that all PCL/MWCNT composites were non-cytotoxic, confirming their suitability for biomedical applications.

## 2. Materials and Methods

### 2.1. Materials

Poly(e-caprolactone) (PCL; Mw ~80,000), multi-walled carbon nanotubes (diameter 6–13 nm, length 6–13 μm, with a purity of ~98%), sulfuric acid (H_2_SO_4_, analytical reagent, 98% purity), and nitric acid (HNO_3_, analytical reagent, 60%) were purchased from Sigma-Aldrich (Naucalpan, Mexico).

### 2.2. Functionalization

To achieve this biocompatibility and their safety for use in biomedical applications, the MWCNT samples were functionalized by refluxing in a 3:1 (*v*/*v*) H_2_SO_4_:HNO_3_ mixture for 8 h prior to use [38,39]. A mixture of concentrated acids and MWCNT was heated under reflux, allowing the vapors to condense and return to the reaction flask. This continuous circulation promotes surface oxidation and the incorporation of functional groups onto the CNT surface [29,30].

### 2.3. Synthesis of PCL Nanofibers with Carbon Nanotubes by the Electrospinning Method

For the electrospinning solution, polycaprolactone (PCL) was dissolved at a concentration of 4.5 wt% in a 1:1 (*v*/*v*) acetone–chloroform solvent mixture, yielding a total solution mass of approximately 340 mg per formulation. Multi-walled carbon nanotubes (MWCNTs) were first dispersed in a 1:1 (*v*/*v*) acetone–chloroform mixture to prepare a stock dispersion, which was sonicated for 1 h to obtain a homogeneous suspension. Controlled aliquots of the CNT stock dispersion were then added dropwise to the PCL solution under ultrasonic agitation to obtain final CNT contents of 0.04, 0.05, 0.075, and 0.2 wt% relative to the PCL mass, corresponding to CNT masses between 0.136 and 0.680 mg, as summarized in Table 1. This dilution-based procedure avoided direct sub-milligram weighing and ensured reproducible CNT incorporation. The selected CNT concentrations (0.04–0.2% *w*/*v*) reflect the high sensitivity of PCL/CNT electrospun systems to small changes in nanotube content and the processing constraints of electrospinning [40,41,42].

Others have demonstrated that the selected concentrations exhibit excellent biocompatibility in biological systems, thereby preserving the material’s functional properties and enhancing the transport of bioactive compounds [43,44]. To avoid inaccuracies associated with direct sub-milligram weighing of CNTs, all nanotube contents were prepared from a pre-weighed stock dispersion of MWCNTs in acetone–chloroform, ensuring analytical precision and reproducibility. The resulting PCL/MWCNT solutions were electrospun using a Fluidnatek LE-100 system (Bioinicia, Valencia, Spain) at a flow rate of 0.9 mL/h, an applied voltage of 10.5 kV, and a tip-to-collector distance of 12 cm, using a flat grounded collector. Ambient temperature and humidity were not controlled [45].

### 2.4. Scanning Electron Microscopy (SEM)

The morphology, tubular structure, and size of MWCNTs, as well as the surface morphology of PCL/MWCNT nanofiber membranes, were characterized using scanning electron microscopy (SEM). Two types of carbon nanotube samples were analyzed: one undispersed, and another sonicated in acetone for 1 h. Both were directly mounted and sputter-coated prior to imaging. Membrane specimens with approximate dimensions of 0.5 cm × 0.5 cm were coated with a thin conductive layer of gold using a Desk IV sputter coater (Denton Vacuum, Moorestown, NJ, USA).

Sputtering was performed in two 60 s cycles at 10 mA to minimize charging and enhance surface conductivity. SEM imaging was conducted using a JSM-6062 (JEOL, Tokyo, Japan). Observations were carried out at accelerating voltages ranging from 10 to 25 kV, with a working distance of 10–15 mm and a spot size of 40–45. The secondary electron imaging (SEI) mode was used to enhance topographical contrast. Images were acquired at various magnifications.

### 2.5. Fourier Transform Infrared Analysis (FT-IR)

The identification of characteristic functional groups and the evaluation of interactions between the polymer matrix (PCL) and the functionalized MWCNTs were analyzed by Fourier-transform infrared spectroscopy (FTIR). The analysis was performed in duplicate using a Cary 630 FTIR spectrophotometer (Agilent Technologies, Santa Clara, CA, USA) equipped with a diamond attenuated total reflectance (ATR) cell and controlled by Agilent Resolutions Pro software (version 5.2.0). Spectra were acquired in transmittance mode over the spectral range of 4000–500 cm^−1^, with a scan rate of 32 scans per sample and a spectral resolution of 2 cm^−1^.

### 2.6. Differential Scanning Calorimetry (DSC)

The thermal properties of the electrospun membranes were analyzed by DSC. Approximately 5 mg of each sample were encapsulated into hermetically sealed aluminum pans, and an empty pan was used as a reference. The analysis was carried out under a nitrogen atmosphere using a DSC-60 Plus (Shimadzu, Kyoto, Japan). Samples were heated from −80 °C to 120 °C at 10 °C/min. The glass transition temperature (Tg), melting temperature (Tm), melting enthalpy (ΔH), and specific heat change (ΔCp) were determined from the resulting thermograms.

### 2.7. X-Ray Photoelectron Spectroscopy (XPS) Analysis

The structural composition of electrospun PCL/MWCNT membranes with varying carbon nanotube concentrations was analyzed by X-ray photoelectron spectroscopy (XPS). For XPS, membrane samples of approximately 1 cm^2^ were analyzed using a Thermo Scientific K-Alpha spectrometer (Thermo Fisher Scientific, Waltham, MA, USA) with an Al Kα source. The measurements were carried out with a pass energy of 200 eV, a spot size of 300 µm, and an energy step size of 1 eV.

### 2.8. Tensile Strength Analysis

#### 2.8.1. Tensile Testing by Dynamic Mechanical Analysis (DMA)

Tensile tests were performed using a dynamic mechanical analyzer (DMA 8000, PerkinElmer, Waltham, MA, USA) operating in stress/strain mode. A controlled load was applied while measuring the material’s deformation response. The tests were conducted at 25 °C, with an initial force of 1 N and a loading rate of 0.1 N/min. Prior to data acquisition, a 1 min preconditioning period was applied, followed by a 5 min isothermal phase to stabilize the system thermally and mechanically.

#### 2.8.2. Atomic Force Microscopy (AFM)

Nanomechanical properties of electrospun PCL membranes containing various concentrations of multi-walled carbon nanotubes (0%, 0.04%, 0.05%, 0.075%, and 0.2%) were evaluated using a Park XE7 Atomic Force Microscope (Park Systems, Suwon, Republic of Korea) in contact mode. Samples (1 cm × 1 cm) were mounted on flat stainless-steel disks to ensure stability during measurements. An SD-R30 CONT 3M probe (NanoSensors, Neuchâtel, Switzerland) was used, suitable for force spectroscopy and nanomechanical analysis.

Measurements were conducted at room temperature using standardized parameters: scan area of 40 µm × 40 µm, resolution of 256 × 256 pixels, Z-scanner speed of 0.2 µm/s, and scan frequency of 0.6 Hz. Deformation depth ranged from 2 to 5 nm to avoid plastic damage. Force–distance spectroscopy was applied, and Young’s modulus was determined by nanoindentation, where the probe applied a perpendicular force at a single point to assess local stiffness via the material’s elastic response under compression.

Force–displacement curves were obtained by approaching and retracting the AFM probe from the sample surface. These curves were analyzed using elasticity theory to calculate the Young’s modulus of the electrospun membranes. The Sneddon model, a refinement of the Hertz model for conical tips, was applied, as it better describes the interaction between the SD-R30 CONT 3M probe and the sample. The analysis assumed predominantly elastic behavior, consistent with the mechanical response of PCL, and minimized adhesion effects due to the 30 nm tip radius.

### 2.9. Cytotoxicity Assessment

Peripheral blood mononuclear cells (PBMCs) were prepared from buffy coats obtained from healthy donors for the blood bank of Instituto Nacional de Enfermedades Respiratorias Ismael Cosío Villegas. The institutional review boards approved the protocol and waived informed consent on the grounds of subject anonymity (code B10-25). One million PBMCs were seeded per well in 24-well ultralow-attachment plates (Costar) and cultured in the presence of medium RMPI-1640 (Gibco, Waltham, MA, USA) supplemented with 2 mM glutamine (Lonza, Basel, Switzerland) and 10% inactivated human serum (Valley Biomedical, Winchester, VA, USA), used here as a negative control, and 1 cm^2^ of the PCL/MWCNT preparations. The cells were cultured for 24 h, 3 days, and 7 days at 37 °C in a 5% CO_2_ atmosphere. The cells were retrieved, and viability was measured by trypan blue exclusion. The cells were counted using a hemocytometer, and the results are reported as a percentage of viability. To account for the effects of culturing for several days, the viability observed in cells incubated only with medium was set at 100%. The impact of PCL/MWCNT contact on cell viability was then reported relative to the untreated cells.

## 3. Results

### 3.1. Scanning Electron Microscopy (SEM) Results

**Figure 1:**
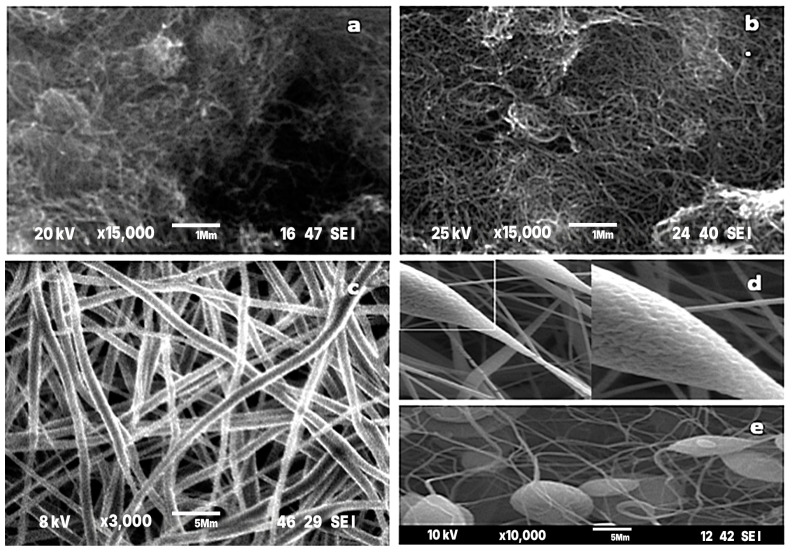
(**a**) CNTs before ultrasonic bath, (**b**) CNTs after ultrasonic bath, (**c**) Membrane of electrospun pure polycaprolactone fibers, (**d**,**e**) PCL nanofibers with the nanotubes embedded.

We first defined the surface morphology of PCL/MWCNT nanofiber membranes. Carbon nanotubes were elongated, cylindrical, intertwined, fibrous-looking structures with diameters under 50 nanometers that weave three-dimensional networks and do not form agglomerations (Figure 1a). The membrane of electrospun polycaprolactone fibers consisted of elongated fibers with a cylindrical geometry and diameters below one micron (Figure 1b). Once the PCL was reinforced with MWCNTs, the nanofibers had diameters below 100 nm interspersed with high-porosity bulbs and microspheres with diameters ranging from 240 nm to 2 μm (Figure 1c).

The presence of microspheres in the nanofibers has previously been explained by the dilatation theory of electrospinning [46], which describes the behavior of the polymer solution under an electric field. This behavior is related to the solution’s viscosity, concentration, and physical and chemical properties, as well as electrostatic forces [47,48]. They could also be explained by the PCL concentration in the solution (4.5% *v*/*v*), the applied voltage (10.5 kV), and the development of the electrospinning process. During the process, the solution droplets were stretched into nanofibers, which then collapsed into microspheres. The porosity could be attributed to the evaporation rates of acetone and chloroform, as well as to environmental conditions such as humidity and temperature [6,49,50].

**Figure 2:**
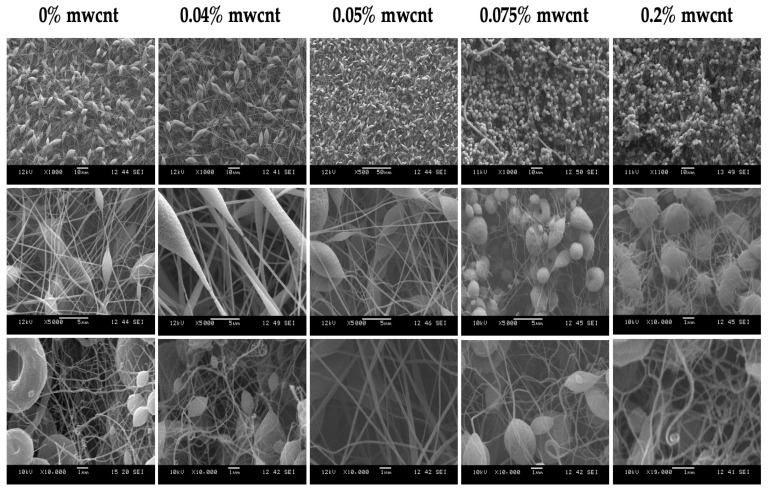
SEM micrographs of electrospun PCL membranes containing different concentrations (0%, 0.04%, 0.05%, 0.075%, 0.2%) of MWCNTs.

The concentration of MWCNTs affected the surface morphology of the PCL preparations (Figure 2). In the absence of MWCNTs, the nanofibers had diameters ranging from 195 nm to 250 nm, appeared long and continuous, and had smooth surfaces. As the concentration of MWCNTs increases (from left to right in the micrographs), a noticeable increase in fiber diameter is observed, reaching up to 395 nm. A similar trend of increasing electrospun fiber diameter with higher CNT content has been reported by others [51]. This behavior suggests enhanced interaction and dispersion of the MWCNTs within the polycaprolactone matrix [52,53], potentially influencing the overall properties of the PCL/MWCNT composite.

Figure 2 with 0% MWCNTs, corresponding to pure polycaprolactone (PCL), exhibits a relatively smooth surface without agglomerates. The nanofibers appear long and continuous, indicating a typical unmodified PCL morphology. In the sample with 0.04% MWCNTs, the structure remains primarily uniform; however, increased porosity is observed in the upper row of images, likely due to the incorporation of a small amount of carbon nanotubes. In the middle and lower rows, PCL nanofibers with low MWCNT content appear to contain embedded nanotubes within them. The sample containing 0.05% MWCNTs exhibits a surface with greater porosity than pure PCL. In the subsequent images (middle and lower rows), the nanofibers appear thicker, and the presence of carbon nanotubes is more evident within the membrane, suggesting stronger interactions between MWCNTs and the PCL matrix [51,52,53].

In the sample with 0.075% MWCNTs, a significantly more porous surface is observed, with a more evident distribution of nanotubes, indicating improved dispersion in the PCL matrix. Although no quantitative porosity values were obtained, qualitative SEM observations indicate increased surface porosity at 0.075% MWCNT, a feature known to play a critical role in the functional performance of electrospun nanofibrous membranes, particularly in drug delivery applications [54]. The nanofibers form a denser, more clustered structure, with a particle-like or micro-sphere appearance, possibly due to MWCNT aggregates. Finally, in the sample containing 0.2% MWCNTs, the micrograph revealed regions with slight nanotube agglomeration and a highly porous morphology. The nanofibers are entangled with these agglomerated areas, which may result from oversaturation of the PCL matrix with carbon nanotubes.

Electrospinning dilation has been studied to control the number of microspheres, their diameter, and porosity, thereby leveraging these characteristics for biomedical applications. Electrospun nanofibers offer several advantages. In tissue engineering, their reduced fiber diameter mimics the extracellular matrix and enhances cell adhesion. Their high porosity improves drug diffusion and influences release rates in drug delivery applications. This fiber geometry also provides mechanical properties like flexibility, elasticity, and stiffness comparable to natural tissues and bone structures. Additionally, their morphology could promote better biological integration, reduce immune responses, and improve biocompatibility [49,55,56]. These structures mimic the extracellular matrix, enhancing cell adhesion, making them ideal for tissue engineering [23]. Furthermore, the resulting membranes exhibit high porosity, allowing for improved diffusion and controlled release of encapsulated drugs. Regarding mechanical properties, these fibers combine flexibility, elasticity, and stiffness, making them similar to natural tissues and bone structures [49]. Due to the carbonaceous nature of both PCL and MWCNTs, EDX provides insufficient elemental contrast to distinguish between phases; therefore, FTIR and SEM were employed as the primary techniques to assess structural interactions and morphology [57,58].

### 3.2. Fourier Transform Infrared Analysis FTIR Results

**Figure 3:**
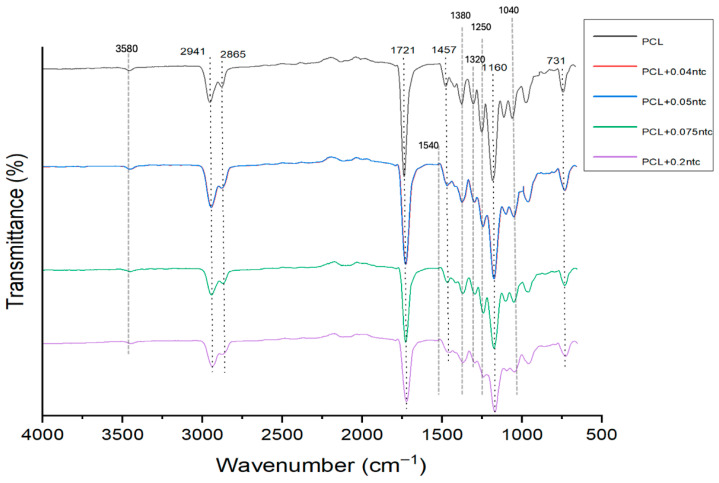
FT–IR spectra of the PCL/MWCNT composite membranes at different concentrations (0%, 0.05%, 0.075%, 0.2%).

The FT-IR spectra of the PCL/MWCNT composite membranes at different concentrations (0%, 0.05%, 0.075%, 0.2%) showed the characteristic peaks of polycaprolactone, including those corresponding to the asymmetric and symmetric stretching vibrations of the aliphatic C-H bonds at ~2941 cm^−1^ and 2850 cm^−1^, respectively (Figure 3). The peak around 1721 cm ^−1^ was attributable to the stretching vibrations of the carbonyl group (C=O). In addition, CH_2_ vibrations were detected at 1457 cm^−1^, the ester group (C-O-C) at 1160 cm^−1^, and the deformation vibrations of the (C-H) bonds in the polymer chains around 731 cm^−1^ [59,60]. The functionalization of the carbon nanotubes caused the appearance of peaks at 2941 cm^−1^ and 2850 cm^−1^, indicating the presence of aliphatic chains introduced during the functionalization of MWCNT through oxidative treatment with concentrated acids such as nitric and sulfuric acid. In addition, the presence of a carboxyl group at 1721 cm^−1^, which overlaps with the carbonyl band of the PCL, confirms the success of the functionalization [61]. The strong interaction between the polymeric matrix of the PCL and the MWCNTs is confirmed by the decrease in the intensity of the band at ~1457 cm^−1^, corresponding to CH_2_, which suggests changes in the mobility of the PCL chains [61,62].

Additionally, the acidic functionalization of the MWCNTs was evidenced by the broadening of the band near 3580 cm^−1^, associated with –OH/–COOH groups introduced during oxidative treatment. A slight shift of the carbonyl peak from 1721 cm^−1^ to 1715 cm^−1^ further suggests hydrogen bond formation between PCL and functionalized nanotubes. The reduction of the band at 1380 cm^−1^, corresponding to C–O stretching of the ester group, and the decreased intensity of the 1320 cm^−1^ band indicate modifications in the crystalline structure of PCL upon nanotube incorporation. Additional signals at 1250, 1160, and 1040 cm^−1^ reflect strengthened intermolecular interactions between MWCNTs and PCL chains. The diminished intensity of the CH_2_ band at ~1457 cm^−1^ also corroborates restricted polymer chain mobility due to nanotube polymer interactions [63,64,65,66].

### 3.3. Tensile Strength Analysis Results

#### 3.3.1. Atomic Force Microscopy (AFM) Results

**Figure 4:**
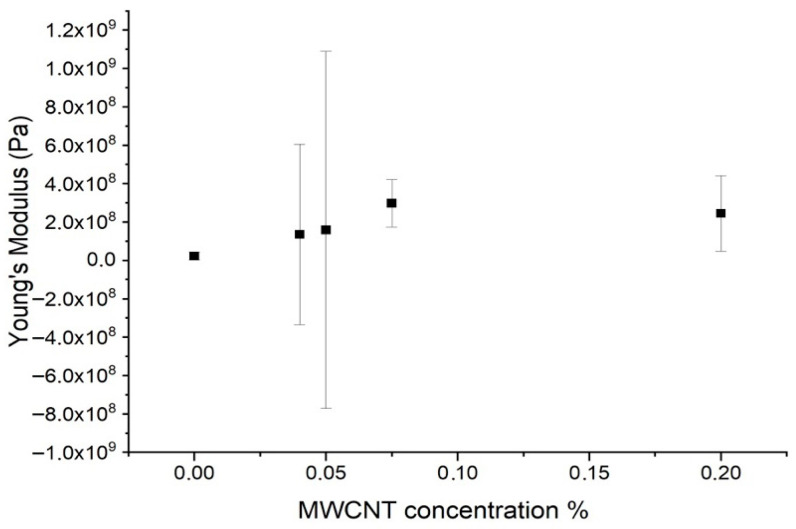
Membranes’ Young’s Modulus obtained by Atomic Force Microscopy (AFM).

Young’s modulus was measured at different points on individual nanofibers using atomic force spectroscopy (Table 2). The recorded values for MWCNT concentrations of 0%, 0.04%, 0.05%, 0.075%, and 0.2% were 22 MPa, 135 MPa, 159 MPa, 298 MPa, and 245 MPa, respectively. As shown in Figure 4, the addition of 0.075% MWCNTs led to the highest stiffness, increasing the Young’s modulus by up to 13.5 times compared to pure PCL. Even at 0.2% MWCNTs, the modulus remained approximately 11 times higher than the unreinforced sample, despite a slight decrease relative to the peak value.

This reduction in Young’s modulus at the higher concentration (0.2%) may be attributed to nanotube agglomeration and microstructural changes induced by the electrospinning jet expansion phenomenon, which can disrupt fiber alignment. Nonetheless, electrospinning expansion may also have promoted partial alignment of MWCNTs, contributing to local mechanical reinforcement. The 18% drop in modulus at 0.2% MWCNTs is also likely related to reduced load-transfer efficiency due to nanotube aggregation, as observed in SEM micrographs [67]. Literature reports that electrospun PCL typically exhibits a Young’s modulus between 10 and 50 MPa [68]. The results of this study show significant improvements due to the effective dispersion and interaction of MWCNTs within the polymer matrix at low concentrations, consistent with previous findings [47,69,70].

The inherent inhomogeneity of electrospun membranes, variations in fiber diameter, the presence of microspheres, and differences in local alignment can explain measurement variability. Additionally, tip-sample interaction and substrate effects are known to influence AFM-based mechanical measurements [71,72].

Overall, the results confirm a clear trend of mechanical reinforcement in PCL/MWCNT nanocomposites, with optimal performance at 0.075% MWCNTs. While electrospinning-induced expansion may limit mechanical improvements at higher concentrations, the MWCNT-PCL interaction remains effective in aligned regions, resulting in increased stiffness relative to poorly aligned regions. Although the mean Young’s modulus appeared to increase with CNT incorporation, the high standard deviations characteristic of AFM measurements on electrospun fibers indicate that these differences are not statistically significant. Thus, the mechanical data should be interpreted as qualitative trends consistent with CNT-induced reinforcement, rather than as statistically distinguishable improvements.

#### 3.3.2. Dynamic Mechanical Analyzer (DMA) Results

**Figure 5:**
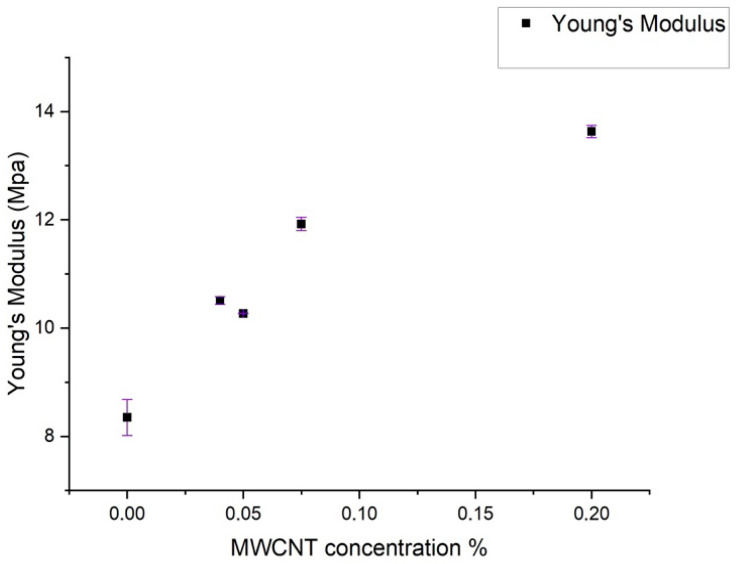
Membranes’ Young’s Modulus obtained by Dynamic Mechanical Analyzer (DMA).

Due to the sensitivity and delicate handling of the electrospun membranes, an alternative technique was selected to determine the Young’s modulus, aiming to reduce the variability in standard deviation values. As shown in Table 3 and Figure 5, this method yielded generally lower modulus values compared to AFM measurements, but with a more consistent standard deviation.

The results also confirm a reinforcement effect that increases with MWCNT concentration. Although higher MWCNT content was associated with agglomerations, morphological defects, and bead formation in the fibers, the significant improvement in membrane stiffness indicates that MWCNTs serve as an effective reinforcement even at low concentrations.

Although SEM micrographs revealed bead defects, fiber discontinuities, and localized MWCNT agglomerates, the consistent increase in modulus across both datasets indicates that MWCNTs act as effective reinforcement even at very low concentrations.

### 3.4. DSC Results

**Figure 6:**
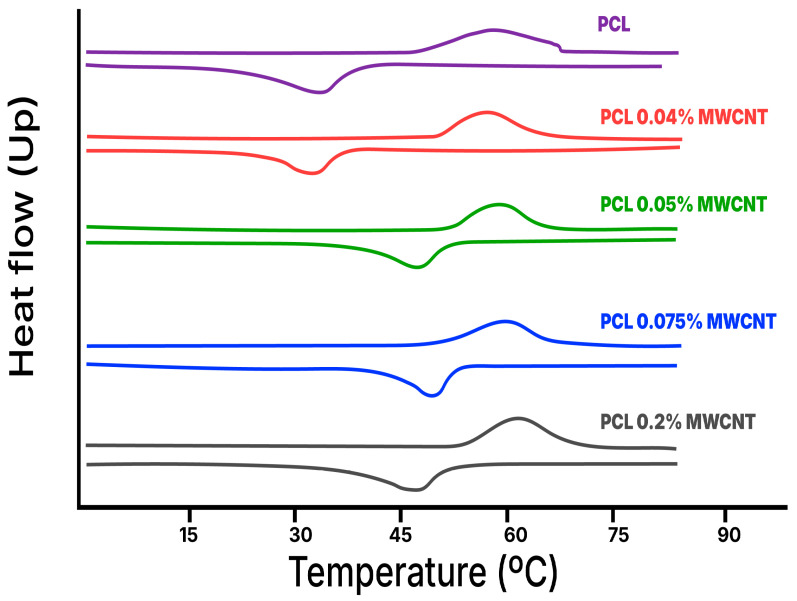
Differential scanning calorimetry (DSC) analysis of the membranes with different MWCNT content.

Differential scanning calorimetry (DSC) analysis of the electrospun membranes revealed a shift in the crystallization temperature (Tc) of PCL upon the incorporation of the functionalized MWCNTs (Figure 6). While the melting temperature (Tm) of PCL remained within its characteristic range (55–60 °C) [73], the Tc increased from approximately 35 °C to 45 °C [74], indicating a nucleating effect induced by the MWCNTs. This effect is attributable to the interaction between the polymer chains and the nanotube surfaces, which facilitates molecular alignment and promotes more efficient crystallization [74,75]. Additionally, the chain orientation generated during the electrospinning process likely contributes to the enhanced crystalline organization. Despite the high thermal stability of MWCNTs in inert environments (up to ~2800 °C), their surface modification and dispersion within the polymer matrix allow them to modulate the crystallization behavior without affecting the thermal degradation range of the composite [76]. The increased Tc, combined with the preserved Tm, suggests improved molecular packing and thermal response, which may correlate with enhanced mechanical rigidity. These thermal modifications highlight the potential of the composite membranes for biomedical applications that require thermal stability and controlled drug delivery, such as for close contact with the skin or dermis.

The stretching effect induced during electrospinning reduces the crystallinity of pure PCL (Table 4). However, as the concentration of MWCNTs increases, they compensate for the amorphous character of the polymer chains by acting as nucleating agents. This results in a progressive increase in both the crystallization temperature and the degree of crystallinity, thereby providing the membranes with enhanced stiffness and structural stability.

### 3.5. XPS Results

**Figure 7:**
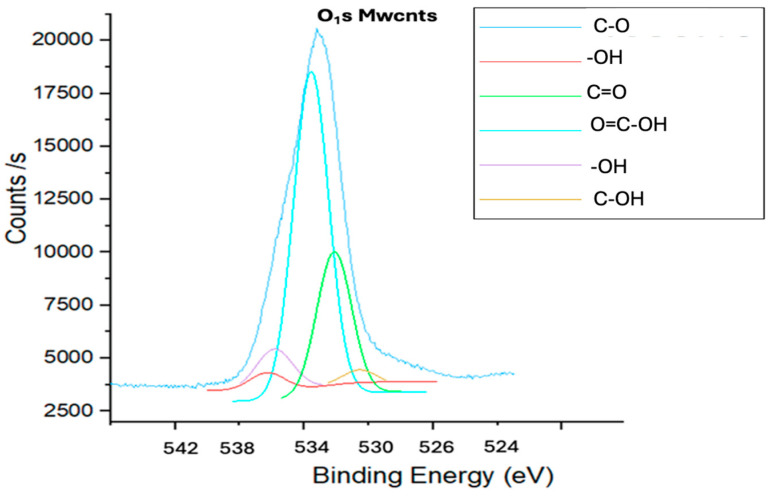
Structural composition of electrospun PCL/MWCNT membranes with different concentrations of carbon nanotubes.

The high-resolution XPS spectrum of the O1s core level reveals the presence of multiple oxygen-containing functional groups within the nanocomposite material (Figure 7). The deconvolution of the O1s peak indicates at least four distinct components, each corresponding to oxygen atoms in different chemical environments. The most intense peak, centered around 532.5–533.5 eV, is typically attributed to C–O bonds such as those found in alcohols or ether groups, suggesting the presence of hydroxylated or ether-like species on the surface of the MWCNTs or within the polymer matrix. A second component, observed near 531.0–531.5 eV, is assigned to carbonyl (C=O) functionalities, indicating the possible formation of ketones, aldehydes, or ester groups.

Another peak at 530.0–530.8 eV can be associated with carboxylic acid groups (O=C–OH), which may result from oxidative functionalization or chemical interactions with the matrix. Finally, a less intense feature near 534 eV may correspond to physically adsorbed water or loosely bound hydroxyl groups.

The presence of these oxygen functionalities confirms the successful chemical modification of the MWCNT surface and suggests strong interfacial interactions between the nanotubes and the surrounding fiber matrix. This surface chemistry likely enhances nanotube dispersion and improves compatibility and potential load transfer within the composite fibers [77,78]

### 3.6. Cytotoxicity Assessment Results

**Figure 8:**
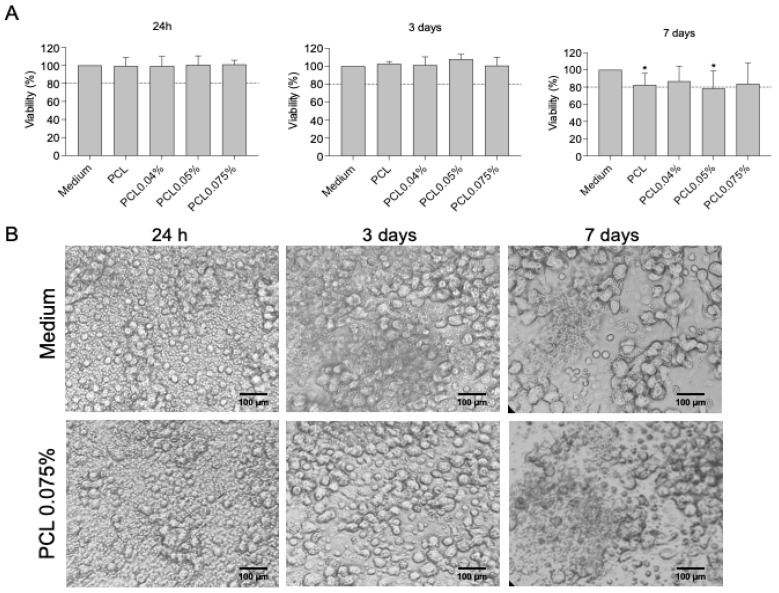
Cytotoxicity of the PCL preparations. Primary human mononuclear cells from healthy subjects were exposed to PCL preparations and cultured for 24 h, 3 days, and 7 days at 37 °C and 5% CO_2_. (**A**) Cell viability was quantified by trypan blue exclusion. Cells incubated in medium only were considered the reference, and their viability was set at 100%. Depicted are the means and standard deviations (n = 6). * *p* < 0.05, Shapiro–Wilk normality test followed by a one-sample *t*-test. (**B**) Representative micrographs of the cell cultures. Depicted are the unstimulated cells (Medium) and the cells exposed to the PCL membranes with the highest concentration of MWCNTs. Scale bars indicate 100 μm.

We evaluated the viability of primary human peripheral blood cells, comprising lymphocytes and monocytes, upon contact with the PCL preparations. We used cells cultured in medium alone as the reference. The cell viability was good at 24 h and 3 days of culture. However, at 7 days, the cell viability was significantly reduced (Figure 8A). For a preparation to be considered safe, cell viability must be over 80%, as indicated in the graphs by a dotted line. Representative images of the cell cultures show the normal development of mononuclear cells over 7 days of culture (medium, Figure 8B), during which monocytes double in size by the seventh day. Despite reduced cell viability by day 7, the PCL preparations are considered non-cytotoxic, as viability ranged within safe parameters [79,80]. Additional evaluations will be necessary when drugs are incorporated, but these preliminary analyses warrant further studies using specific drugs.

## 4. Conclusions

In this study, electrospun polycaprolactone (PCL) nanofibrous membranes reinforced with functionalized multi-walled carbon nanotubes (MWCNTs) were successfully synthesized and characterized for potential biomedical applications. The incorporation of MWCNTs resulted in significant improvements in mechanical and thermal performance. Spectroscopic analyses (XPS and FTIR) confirmed the effective integration of the nanotubes into the polymeric matrix, while mechanical and crystallization studies demonstrated their reinforcing and nucleating effects.

Biological assays using peripheral blood mononuclear cells (PBMCs) showed the non-cytotoxicity of the electrospun membranes. Overall, PCL/MWCNT nanofibrous membranes are promising multifunctional wound dressings and transdermal drug delivery systems. Future research will focus on evaluating the controlled release of therapeutic agents, long-term biocompatibility, and the in vivo performance of these nanocomposite membranes to further validate their biomedical applicability.

## Figures and Tables

**Table 1:** MWCNT concentrations and electrospinning parameters for the membrane’s synthesis.

PCL Concentration mg	CNTs %	CNTs mg	Total Weight mg	Voltage kV	Flow Volume mL/h	Distance cm
340.06	0.04	0.13608	340.2	10.5	0.9	12
340.02	0.05	0.1701	340.2	10.5	0.9	12
339.94	0.075	0.2551	340.2	10.5	0.9	12
339.51	0.2	0.6804	340.2	10.5	0.9	12

**Table 2:** AFM Young’s Modulus of the membranes with different MWCNT concentrations.

MWCNT’s Concentration	0%	0.04%	0.05%	0.075%	0.2%
Young’s Modulus	22 MPa	135 MPa	159 MPa	298 MPa	245 MPa
Standard deviation	19.8 MPa	470 MPa	931 MPa	1.24 GPa	197 MPa

**Table 3:** DMA Young’s Modulus of the membranes with different MWCNT concentrations.

MWCNT Concentration	0%	0.04%	0.05%	0.075%	0.2%
Young’s Modulus (MPa)	8.54	10.51	10.27	11.92	13.63
Standard deviation (MPa)	3.31	0.0705	0.00363	0.1225	0.11202

**Table 4:** Enthalpy of crystallization.

Sample	Tc (°C)	|∆Hc(J/g)|	X_C_ (%)
PCL	34.3	30	21.50
PCL 0.04% MWCNT	34.5	40	28.86
PCL 0.05% MWCNT	47.2	40	28.86
PCL 0.075% MWCNT	45.2	50	35.84
PCL 0.2% MWCNT	44.9	60	43.09

## Data Availability

Data is contained within the article.
